# Connexin-43 upregulation in micrometastases and tumor vasculature and its role in tumor cell attachment to pulmonary endothelium

**DOI:** 10.1186/1741-7015-6-20

**Published:** 2008-07-22

**Authors:** M Khair Elzarrad, Abu Haroon, Klaus Willecke, Radoslaw Dobrowolski, Mark N Gillespie, Abu-Bakr Al-Mehdi

**Affiliations:** 1 Department of Pharmacology and Center for Lung Biology, University of South Alabama, North University Boulevard, Mobile, AL 36688, USA; 2 University of Bonn, Institute for Genetics, Roemerstrasse, 53117 Bonn, Germany

## Abstract

**Background:**

The modulation of gap junctional communication between tumor cells and between tumor and vascular endothelial cells during tumorigenesis and metastasis is complex. The notion of a role for loss of gap junctional intercellular communication in tumorigenesis and metastasis has been controversial. While some of the stages of tumorigenesis and metastasis, such as uncontrolled cell division and cellular detachment, would necessitate the loss of intercellular junctions, other stages, such as intravasation, endothelial attachment, and vascularization, likely require increased cell-cell contact. We hypothesized that, in this multi-stage scheme, connexin-43 is centrally involved as a cell adhesion molecule mediating metastatic tumor attachment to the pulmonary endothelium.

**Methods:**

Tumor cell attachment to pulmonary vasculature, tumor growth, and connexin-43 expression was studied in metastatic lung tumor sections obtained after tail-vein injection into nude mice of syngeneic breast cancer cell lines, overexpressing wild type connexin-43 or dominant-negatively mutated connexin-43 proteins. High-resolution immunofluorescence microscopy and Western blot analysis was performed using a connexin-43 monoclonal antibody. Calcein Orange Red AM dye transfer by fluorescence imaging was used to evaluate the gap junction function.

**Results:**

Adhesion of breast cancer cells to the pulmonary endothelium increased with cancer cells overexpressing connexin-43 and markedly decreased with cells expressing dominant-negative connexin-43. Upregulation of connexin-43 was observed in tumor cell-endothelial cell contact areas *in vitro *and *in vivo*, and in areas of intratumor blood vessels and in micrometastatic foci.

**Conclusion:**

Connexin-43 facilitates metastatic 'homing' by increasing adhesion of cancer cells to the lung endothelial cells. The marked upregulation of connexin-43 in tumor cell-endothelial cell contact areas, whether in preexisting 'homing' vessels or in newly formed tumor vessels, suggests that connexin-43 can serve as a potential marker of micrometastases and tumor vasculature and that it may play a role in the early incorporation of endothelial cells into small tumors as seeds for vasculogenesis.

## Background

Intercellular communication via gap junctions between neighboring cells contributes to the organization of cells into functional tissues. Malignant tumors arising from planar epithelial tissue lose the normal growth limit caused by cell-cell contacts and form solid tumors in three dimensions. This observation was the origin of the notion that the loss of gap junctional intercellular communication (GJIC) plays an important role in tumorigenesis [1,2]. Moreover, since invasive tumors are characterized by cell detachment from the primary mass, a disruption of intercellular junctions also became a *de facto *requirement for metastasis [3,4].

A tumor cell undergoes a variety of stages in its life cycle: its origin and growth in the primary site, cellular detachment and intravasation, dissemination, settling in a new site by endothelial attachment, and its growth as a metastatic tumor including the development of its blood supply. While some of these stages, such as uncontrolled cell division and cellular detachment, are associated with loss of intercellular junctions, other stages, such as intravasation, endothelial attachment, and vascularization, demand increased cell-cell contact. Therefore, it is clear that a simplistic notion of the loss of cellular communication cannot fully describe the status of the cellular interaction between tumor cells or between endothelial and tumor cells during the whole metastatic process. An adhesive interaction of tumor cells to endothelial cells during 'homing' is obvious. In addition, a role for connexins in the facilitation of tumor growth, metastatic potential, and tumor vascularization has been documented [5-12].

We have demonstrated that endothelial cells are incorporated early in breast cancer metastatic tumors in the lung before the onset of hypoxia and they grow in mixed fashion among the tumor cells, contributing to tumor vascularization. These disparate cell types were shown to establish functional GJIC both *in vivo *and *in vitro*. Since the role of gap junctions in tumorigenesis and metastasis has been controversial as outlined above, we addressed the following questions in this work.

1. Does connexin-43 (Cx43) mediate GJIC between 4T1-GFP cells and pulmonary microvascular endothelial cells (PMVECs)?

2. Does Cx43 play a role in the attachment of breast cancer cells to endothelial cells in the pulmonary circulation?

3. Is Cx43 upregulation associated with the sites of micrometastatic foci and metastatic tumor vasculature?

To understand the role of Cx43 in the metastatic process, we used cancer cell lines that overexpress wild-type Cx43, express a dominant-negative mutant of Cx43, and a control mutant that does not affect endogenous Cx43 functions. Using these Cx43 mutant cell lines, we show that in a syngeneic mouse breast cancer experimental metastasis model, Cx43 enhances breast cancer cell attachment to the pulmonary endothelium. The results also indicate that Cx43 can serve as a marker of micrometastases and tumor vasculogenesis because of its significant upregulation in areas of tumor cell-endothelial cell contact areas *in vitro *and *in vivo*.

## Methods

### Cell culture and stable expression of Cx43 mutants

Pulmonary microvascular endothelial cells were obtained from the Endothelial Cell Culture Core at the Center for Lung Biology [13]. These cells are routinely obtained by primary isolation from Sprague-Dawley rats and are used within passage 12. These cells are maintained and propagated in Dulbecco's Modified Eagle's Medium (DMEM) with 10% fetal bovine serum.

The 4T1 cells (a mouse breast adenocarcinoma cell line) were obtained from ATCC and were transfected with enhanced green fluorescent protein (eGFP) vector (Clontech, Mountain View, CA). Cells with stable GFP expression were separated by fluorescence activated cell sorting and propagated and maintained in DMEM with 10% fetal bovine serum. We have used the 4T1-GFP cells previously in both experimental and spontaneous metastasis assays [14-16]. Cells were grown in DMEM supplemented with 10% fetal bovine serum, 100 units/ml penicillin and 100 mg/ml streptomycin and were maintained at 37°C in a moist environment of 95% air and 5% CO_2 _and sub-cultured as required.

The Cx43 missense mutation G138R was made by site-directed mutagenesis by changing the glycine to arginine on position 138 in the amino acid sequence. The G138R mutant expresses a mutant Cx43 that exhibits a dominant-negative effect when expressed in cells with endogenous Cx43 [17]. This mutation is known to be present in patients suffering from oculodentodigital dysplasia [18,19]. The expressed Cx43-G138R protein translocates to the membrane, and assembles as connexons, but does not exhibit functional GJIC. Cells expressing the G138R mutation exhibited a strong suppression of dye-transfer even when they were co-expressed with wild-type Cx43 [17]. The G138R construct was ligated to a eukaryotic expression vector containing the zeocin resistance gene driven by the SV40 promoter. On the other hand, the C61S mutant Cx43 protein lacks gap junction channel forming abilities because of its cytoplasmic localization, and apparently it does not interfere with functional gap junction formation by endogenous normal Cx43 [20]. The Cx43 point mutation C61S was generated by site-directed mutagenesis, replacing the cysteine residue in position 61 with a serine residue. The C61S construct was primarily used as a control. Both the C61S construct and the cDNA for wild-type Cx43 (Cx43 overexpressors, or Cx43OE) were ligated into the expression vector pBEHpac18. 4T1-GFP tumor cells were also transfected with the vector expressing two copies of wild-type Cx43 (Cx43OE) to enhance the formation of functional gap junctions in tumor cells (positive control).

Plasmid DNA was extracted from filter paper spots into 500 μl of Tris-EDTA buffer (10 mM Tris-Cl, pH 7.5 and 1 mM EDTA), electroporated into competent bacteria, and allowed to grow overnight with the appropriate antibiotic for selection. The plasmid DNA was isolated using Miniprep (Qiagen) and samples were run on a gel to confirm the size of the plasmid. In addition, samples of the plasmid DNA were sent to the GeneLab at Louisiana State University (Baton Rouge, LA) for amplification and sequencing. The presence of each mutation was confirmed by comparison with wild type using BLAST software.

4T1-GFP cells were grown to 75–95% confluency in 60 mm culture dishes and transfected at 37°C in Opti-MEM medium (Invitrogen, Carlsbad, CA) containing 75 μl of Lipofectamine 2000 and 30 μg of plasmid DNA. After 24 hours, the DNA/Lipofectamine suspension was removed and replaced with culture medium containing the appropriate antibiotics. Cells were selected with 2 μg/ml puromycin (InvivoGen, San Diego, CA) for the C61S and Cx43OE constructs or with 500 μg/ml of zeocin (Invitrogen) for the G138R construct. The selection process continued for 2 weeks on the puromycin resistance constructs and for 4 weeks on the zeocin resistant construct. Cells were maintained afterwards in normal medium containing the appropriate amounts of antibiotics.

### Evaluation of gap junctions by dye-transfer assays *in vitro *and *in situ*

The presence of functional gap junctions was evaluated using a fluorescent dye-transfer assay. Sub-confluent monolayers of PMVECs in glass-bottomed 35 mm dishes were used. Endothelial cells were washed with Kreb's Ringer bicarbonate solution (KRB), with 5% clinical grade dextran and 10 mM glucose at pH 7.4, and incubated with 5 μM Calcein Orange Red AM (gap junction-permeable fluorescent dye, molecular weight 790 Da) in KRB for 30 minutes. The acetoxymethyl ester (AM) group of the dye is hydrolyzed by esterases in a viable cell, rendering the dye polarized and trapped within the cell, but allowing its transfer to adjacent cells via functional gap junctions. After dye loading, the endothelial cells were washed five times to remove the free dye from the medium. Experiments were performed in KRB in the absence of serum. Then 4T1-GFP cells (transferred-dye acceptors) were placed on the labeled endothelial cells (dye donors) at a ratio of 1:500 (acceptor:donor). After incubating for up to 3 hours at 37°C, the cells were washed and visualized using a Nikon TE2000 epifluorescence microscope. For *in situ *dye-transfer studies, 5 μM Cell Tracker Orange was used to label pulmonary endothelial cells *in situ *and then Calcein Green AM labeled 4T1 cancer cells (without GFP) were administered in the perfusate of mouse lungs and dye transfer to endothelial cells *in situ *was evaluated by confocal microscopy (BioRad Radiance 2000, Hercules, CA). All fluorescent probes were obtained from Invitrogen Molecular Probes (Eugene, OR).

### Immunocytofluorescence for Cx43

Following stable transfection with wild-type or mutant DNA constructs, cells were grown on glass-bottomed 35 mm dishes and were fixed in ice-cold 70% methanol and 20% acetone solution for 10 minutes at 4°C, then rinsed with phosphate buffered saline (PBS) and prepared for immunolabeling. Non-specific antigen sites were blocked with 2% bovine serum albumin (BSA) in PBS, immunolabeled with the Cx43 primary antibody, washed with PBS and labeled with a fluorescent secondary antibody. Cells were then washed in PBS, air-dried, and cover-slipped with a drop of the mounting medium Gold Antifade Reagent containing 4',6-diamidino-2-phenylindole (DAPI) for nuclear staining (Invitrogen, Carlsbad, CA).

### Western blot analysis and densitometry

Lysates of 4T1-GFP cells collected from stable colonies after transfection with 30 μg of wild-type or mutant cDNA encoding C61S or G138R were subjected to Western blotting. Blots were immunolabeled for Cx43 using a mouse anti-Cx43 monoclonal antibody (catalog #13-8300, Zymed, San Francisco, CA). Unsaturated blots were scanned and densitometry was performed using Quantity One software (BioRad, Hercules, CA).

### Immunohistochemistry/immunohistofluorescence

Immunohistochemistry was carried out on paraffin-embedded mouse lung tissue sections containing tumor segments. Sections were deparaffinized in two changes of xylene then hydrated in sequential changes of decreasing concentrations of ethanol (100%, 90%, 70%, 50%, 30%) followed by a rinse in distilled water. Antigen retrieval was performed by immersing the slides in sodium citrate buffer (10 mM sodium citrate, 0.05% Tween 20, pH 6.0) at boiling temperature. Slides were then cooled at room temperature for 20 minutes and rinsed in PBS buffer containing 0.1% Triton X-100. Nonspecific binding was blocked using 5% goat serum and 5% BSA and 0.3% Triton X-100 for 30 minutes. Slides were incubated with mouse anti-Cx43 monoclonal antibody (#35-5000, Zymed, San Francisco, CA) for 2 hours at room temperature. Slides were rinsed again and incubated for 1 hour with a secondary antibody conjugated to a fluorescent probe (Alexa Fluor 647) or horseradish peroxidase (HRP). Slides were then washed in PBS, air-dried, and cover-slipped with a drop of the mounting medium Gold Antifade Reagent containing DAPI for nuclear staining (Invitrogen, Carlsbad, CA).

### Quantification of tumor cells adhering to the lung vasculature

Immunohistofluorescence and immunohistochemistry slides were imaged using the Nikon TE2000 epifluorescence microscope with a ×60 objective. Three lungs from animals containing tumors derived from each cell line were used. Four slides from each lung were used for imaging. Twenty contiguous picture frames per slide were acquired. Cells were counted in each stitched meta-picture.

### Fluorescence microscopy

A high-resolution digital fluorescence video imaging system based on a Nikon TE-2000 inverted microscope was used. The imaging system is fitted with two automated 10-position filter wheels for both excitation and emission (Sutter Instruments, model Lambda 10-2), an automated dichroic filter cube changer (Nikon), XYZ axis mechanized stage (Prior Scientific, Inc.), a high-resolution 12-bit Orca-100 ER IEEE1394 digital camera (Hamamatsu Inc.), MetaMorph 7.5 image acquisition, processing, and analysis software with three-dimensional reconstruction and point-spread function-based deconvolution capabilities (Universal Imaging/Molecular Devices). Separately addressable excitation, dichroic, and emission filters allow the maximum flexibility for multiple wavelength and ratio imaging in this system.

For imaging metastatic tumors of GFP-expressing 4T1 cells *in situ *in the intact mouse lung, an established isolated lung microscopy method was utilized [15,21]. Some isolated lungs taken from animals with experimental metastases were perfused with Griffonia simplicifolia lectin conjugated with TRITC (Molecular Probes, Eugene, OR) via the pulmonary artery for 30 minutes to label the pulmonary and the tumor microvasculature. The lungs were placed in a specially designed Plexiglas chamber over a coverslip-window at the bottom with the posterior surface of the lung gently touching the coverslip. Subpleural tumors were directly visualized at high magnification (×600) by epifluorescence microscopy using appropriate filters and illumination. Image processing and analysis were performed with MetaMorph software (Molecular Devices, Sunnyvale, CA).

### Experimental metastasis model

A syngeneic breast cancer experimental metastasis model was used in the study as described previously [22]. The 4T1-GFP cell line was derived from the 4T1 mouse mammary adenocarcinoma cell line (# CRL-2539, ATCC, Manassas, VA) by stable transfection with eGFP (Clontech, Mountain View, CA). We injected young nu/nu mice (Charles River) into the tail-vein with single-cell suspensions of 4T1-GFP cells at 30 × 10^3 ^cells per gram body weight followed by lung isolation, perfusion, and intact organ epifluorescence microscopy up to 21 days after injection. The volume of injected medium with cells was 100 μl. Animals were monitored on a daily basis for signs of respiratory distress. The research was conducted under a protocol reviewed and approved by the University of South Alabama Institutional Animal Care and Use Committee.

### Statistical analysis

Data analysis was performed using SigmaStat (Systat Software Inc., San Jose, CA) using one-way analysis of variance and Bonferroni's test. Data are expressed graphically as means ± standard error of the mean (SEM) using SigmaPlot (Systat Software Inc., San Jose, CA). Differences were considered significant with *P *< 0.05.

## Results

### Cx43 expression in tumor cell-endothelial cell co-culture *in vitro *and in pulmonary metastasis *in vivo*

#### Cx43 is upregulated in tumor cell-endothelial cell contact areas

**Figure 1:**
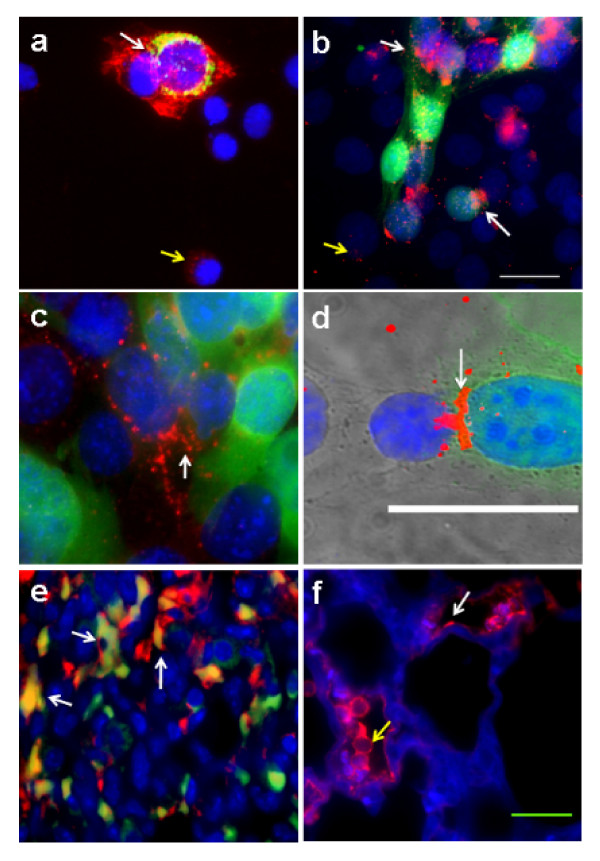
**Upregulation of connexin-43 in tumor cell-endothelial cell contact areas *in vitro *and *in vivo***. (a)-(d) Immunocytofluorescence detecting connexin-43 (Cx43; red) in co-culture of 4T1-GFP cells (green cytoplasmic staining with blue nuclei) and pulmonary microvascular endothelial cells (PMVECs; blue nuclei without cytoplasmic staining). 4T1-GFP and PMVECs were plated together in co-culture and examined under the green fluorescent protein (GFP) channel (4T1 cells), UV-channel (nuclei), and Cy5-channels (Cx43), and shown as an overlay of all channels indicating the location of 4T1-GFP cells (green), nuclei of both cell types (blue), and Cx43 (red). In (a), (b), and (c), upregulation of Cx43 expression (red) is seen at the contact areas between the tumor cells and PMVECs (white arrows). Cells not in a heterologous contact show little expression of Cx43 (yellow arrows). Panel (d) shows an additional overlay of the DIC image to further emphasize the Cx43 intense expression at the areas of contacts (white arrow) between an endothelial cell on the left and a 4T1-GFP cell on the right. (e) Immunohistofluorescence of a lung metastatic tumor section stained for both Cx43 (red) and CD31 (green) with nuclear DAPI staining in blue. Most endothelial cells within the tumor mass (green) showed an increase in Cx43 staining (arrows). (f) Immunohistofluorescence of a fixed mouse lung section after 12 hours of tail vein injection of tumor cells. Autofluorescence of lung tissue in the GFP-channel and the GFP fluorescence of 4T1-GFP cells were assigned the blue pseudocolor for better contrast with the Cy5-channel Cx43 signal (red). Tumor cells can be seen lodged inside pulmonary microvessels. The image clearly indicates the upregulation of Cx43 in both the endothelial cells (white arrow) and the tumor cells within the vessels adhering to the vessel walls (yellow arrow). The scale bar in (a)-(d) is 10 μm and (e), (f) is 25 μm.

Co-culture of 4T1-GFP cells (green cytoplasmic staining with blue nuclei) and PMVECs (blue nuclei without cytoplasmic staining) lead to upregulation of Cx43 expression (red) at the contact areas between the tumor cells and endothelial cells (Figure 1a, b, c, white arrows). Cells not in a heterologous contact demonstrate baseline expression of Cx43 (Figure 1a and 1b, yellow arrows). An overlay of the differential interference contrast (DIC) image demonstrates the intense expression of Cx43 in the contact areas (white arrow) between an endothelial cell on the left and a 4T1-GFP cell on the right (Figure 1d). Immunohistofluorescence of a lung metastatic tumor section stained for both Cx43 (red) and for CD31 (green) with nuclear DAPI staining in blue demonstrates that more than 95% of endothelial cells within the tumor mass (green) have an increase in Cx43 staining (Figure 1e, arrows). Immunofluorescence of a fixed mouse lung section after 12 hours of tail vein injection of tumor cells depicts tumor cells lodged inside pulmonary microvasculature with concomitant upregulation of Cx43 in both the endothelial cells (Figure 1f, white arrow) and the tumor cells (Figure 1f, yellow arrow).

#### Upregulation of Cx43 marks the sites of micrometastatic foci and metastatic tumor vasculogenesis

**Figure 2:**
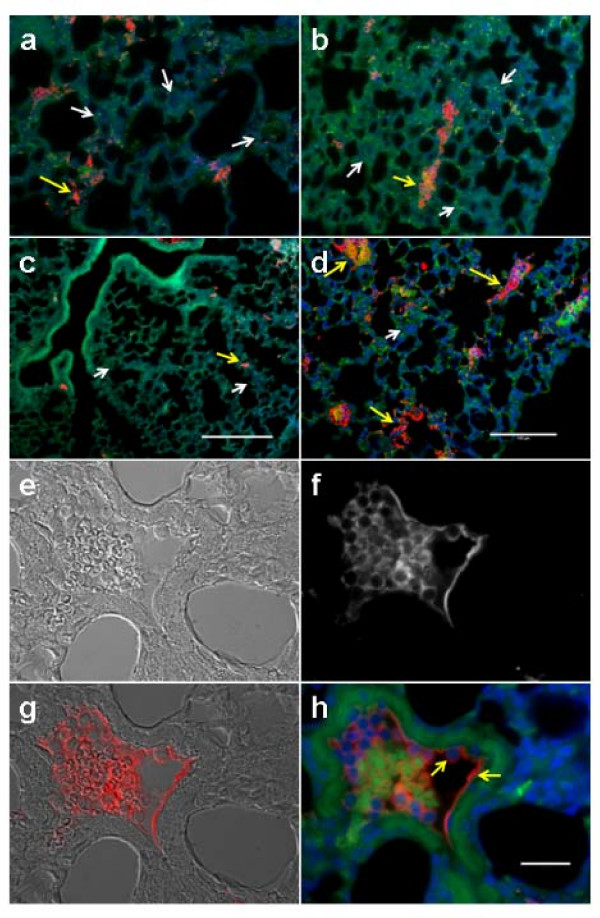
**Connexin-43 expression marks the sites of micrometastases**. (a)-(d), (h) Immunohistofluorescence fixed sections of mouse lung after 12 hours of tail vein injection of 4T1-GFP cells or connexin-43 (Cx43) mutant variants C61S, G138R, or Cx43OE. Autofluorescence of lung tissue and 4T1-GFP fluorescence (green fluorescent protein (GFP) channel green color) and Cx43 signal (Cy5-channel red color), and nuclear staining (DAPI channel blue color) were overlaid. Panels (a)-(d) show images of lung sections taken at low magnification. Panel (a) shows the sections from a lung after tail vein injection of control 4T1-GFP tumor cells. Cx43 (red) was upregulated in vessels containing tumor cells (yellow arrow), while vessels not containing tumor cells from the same lung had barely detectable Cx43 signal (white arrows). Lung sections with C61S expressing tumor cells exhibited similar pattern of Cx43 expression (b), whereas those with the dominant-negative G138R expressing cells exhibited decreased staining of Cx43 (c). In contrast, in lung sections with Cx43 overexpressing tumor cells, a significant increase in the number of intravascular tumor cells, an increase in the number of vessels containing adherent tumor cells, and induction of Cx43 was seen (d). Most of the vessels with Cx43 staining appeared to contain tumor cells (yellow arrows). Panels (e)-(h) show higher-magnification images of lung sections containing tumor cells overexpressing Cx43. Panel (e) shows a DIC image of a vessel within the lung containing tumor cells; (f) shows the Cx43 signal in the same vessel; and (g) is an overlay showing the induction of Cx43 in both endothelium and tumor cells in red color. Panel (h) shows another overlay of the same image of the green (tumor cells, autofluoresence of lung tissue and red blood cells), blue (nuclei), and red (Cy5 for Cx43) channels. Scale bars in (c), (d) are 100 μm (the same scale is used in (a) and (b)) and the scale bar in (h) is 25 μm (the same scale is used in (e), (f), and (g)).

Figure 2 demonstrates that Cx43 expression marks the sites of micrometastases in sections from lungs after tail vein injection of tumor cells. Cx43 (red) was upregulated in vessels containing control 4T1 tumor cells (Figure 2a, yellow arrow), while vessels not containing tumor cells in the same section had barely detectable Cx43 signals (Figure 2a, white arrows). Lung sections with C61S expressing tumor cells exhibited similar pattern of Cx43 expression (Figure 2b), whereas those with the dominant-negative G138R expressing cells exhibited decreased staining for Cx43 (Figure 2c). In contrast, in lung sections with Cx43 overexpressing tumor cells, a significant increase in the number of intravascular tumor cells, an increase in the number of vessels containing adherent tumor cells, and induction of Cx43 were seen (Figure 2d). Ninety five percent of the vessels with Cx43 staining appeared to contain tumor cells (Figure 2d, yellow arrows). Upregulation of Cx43 in tumor cell-endothelial cell contact areas in one of the larger vessels is shown using DIC (Figure 2e), Cx43 signal (white, Figure 2f), overlay of DIC and Cx43 signal (Figure 2g) and overlay of GFP (tumor cells, green), DAPI (nuclei, blue), and Cx43 (red, yellow arrows) signals (Figure 2h).

**Figure 3:**
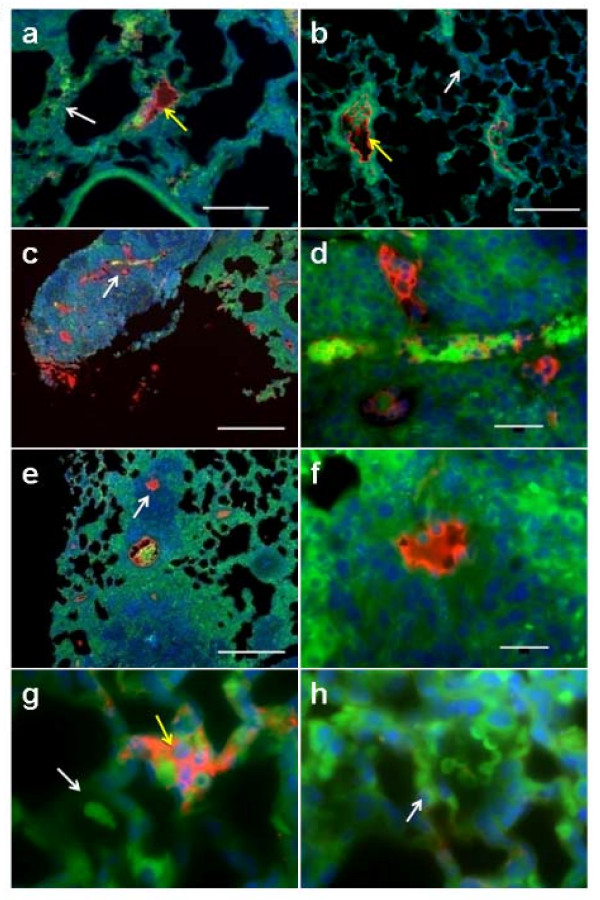
**Intratumor vessels and metastatic foci demonstrate increased connexin-43**. Immunohistofluorescence for connexin-43 (Cx43) of mouse lung sections 12 days after tail vein injection of 4T1-GFP control cells or of cells expressing Cx43 variants C61S, G138R, or Cx43OE. Lung tissue autofluorescence and tumor cell green fluorescent protein (GFP) fluorescence is pseudocolored green color and the Cx43 signal (Cy5-channel) is shown in red. Nuclear staining with DAPI is shown in blue. Each picture is an overlay of these three channels. (a), (b) Lower-magnification images of lung sections containing control 4T1-GFP tumor cells. Cx43 (red) was upregulated in the vessels that contained tumor cells (yellow arrow) while vessels without tumor cells in them had minimal Cx43 signal (white arrows). (c) A metastatic tumor in the lung with enriched Cx43 signal associated with the tumor vasculature. (d) A higher-magnification image of the same tumor highlighting the region with the high Cx43 signal. (e) Another metastatic tumor with (with a higher-magnification image in (**f**)) demonstrating the Cx43 upregulation within tumor vessels. (g) One vessel containing tumor cells and exhibiting high Cx43 signal (yellow arrow), while a neighboring vessel with a stack of red blood cells (white arrow) but no tumor cells shows no Cx43 reactivity. (h) A vessel from a control lung with non-immune mouse IgG used in place of the primary antibody showing no staining of a vessel (arrow) in which red blood cells can be seen due to their autofluorescence in the GFP channel. The scale bar in (a), (b), (c), and (e) is 100 μm and the scale bar in (d) and (f) (which are to the same scale as (g) and (h)) is 25 μm.

Intratumor vessels and metastatic foci demonstrate increased Cx43, making Cx43 a potential marker for not only micrometastases, but also for tumor vasculature (Figure 3). Figures 3a and 3b depict lower-magnification images of lung sections containing control 4T1-GFP tumor cells. Cx43 (red) was upregulated in the vessels that contained tumor cells (yellow arrow) while vessels without tumor cells in them had minimal Cx43 signals (white arrows). A metastatic tumor in the lung with a high level of Cx43 expression in the tumor vasculature is visible in Figure 3c (arrow). Figure 3d shows a higher-magnification image of the same tumor highlighting the region with the high Cx43 signal. Another metastatic tumor at low magnification (Figure 3e) demonstrates Cx43 upregulation within tumor vessels at higher magnification (Figure 3f). Figure 3g shows a vessel containing tumor cells and exhibiting strong Cx43 signal (yellow arrow), while a neighboring vessel with a stack of red blood cells (white arrow) but no tumor cells shows no Cx43 reactivity. A vessel from a control lung treated with non-immune mouse IgG instead of the primary antibody for Cx43 showed no staining of a vessel (arrow) in which red blood cells can be seen due to their autofluorescence in the GFP channel (Figure 3h).

### Role of Cx43 in tumor cell-endothelial cell interaction *in vitro*

#### Cx43 mediates border-sharing and co-growth of 4T1-GFP cells and PMVECs

**Figure 4:**
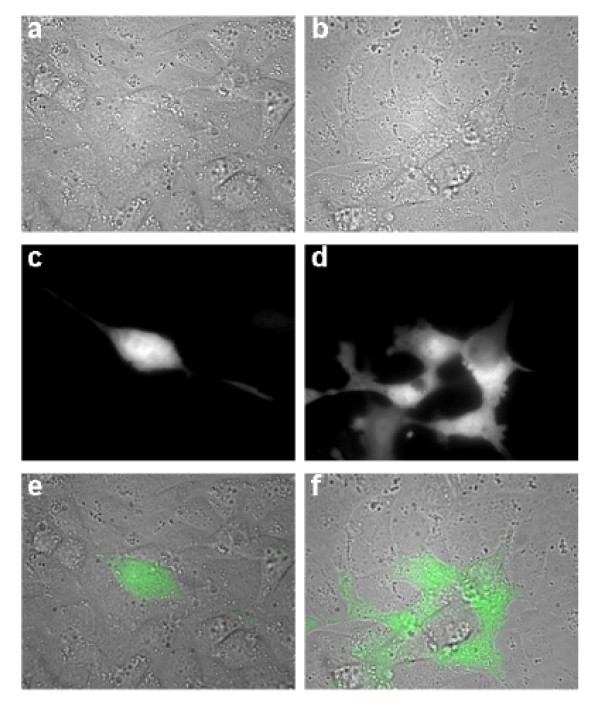
**Tumor cells and endothelial cells share common borders *in vitro***. (a), (b) 4T1-GFP and pulmonary microvascular endothelial cells were plated together in co-culture and examined by DIC imaging. (c), (d) The same fields were imaged in the green fluorescent protein (GFP) channel to reveal the presence of the 4T1-GFP cells. (e), (f) The images were overlaid to show the location and shared borders between the tumor cells and the endothelial cells.

**Figure 5:**
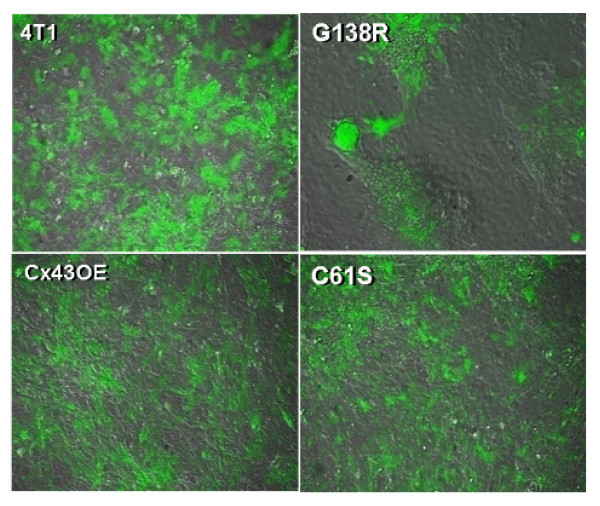
**Patterns of co-growth of 4T1-GFP breast cancer cells (in green) and pulmonary microvascular endothelial cells (DIC channel, gray) *in vitro***. Co-culture of wild-type 4T1-GFP cells (4T1), Cx43-C61S mutation expressing 4T1-GFP cells (C61S), and connexin-43 overexpressing cells (Cx43OE), and pulmonary microvascular endothelial cells exhibited side-by-side growth and shared borders. In contrast, 4T1-GFP cells expressing the mutant Cx43-G138R (G138R) grew on top of endothelial cells without integration into the monolayer.

In co-culture, 4T1-GFP and PMVECs form a monolayer where the cancer cells and endothelial cells could not be identified in DIC pictures (Figure 4A-a,b), but their mixed growth can be ascertained by overlaying the cancer cell pictures in the GFP channel (Figure 4A-c,d) with the DIC pictures (overlays shown in Figure 4A-e,f). Co-culture of wild-type 4T1-GFP, C61S, or Cx43OE and PMVECs exhibited the mosaic growth pattern and the presence of shared borders (Figure 5). In contrast, 4T1-GFP cells harboring the mutant G138R grew on top of endothelial cells without integration into the endothelial monolayer (Figure 5), indicating a possible lack of intercellular communication between the two cell types. This suggests that defective gap junctional communication results in inhibition of coordination required for border-sharing and co-growth.

#### Cx43 mediates establishment of functional GJIC between 4T1-GFP cells and PMVECs

**Figure 6:**
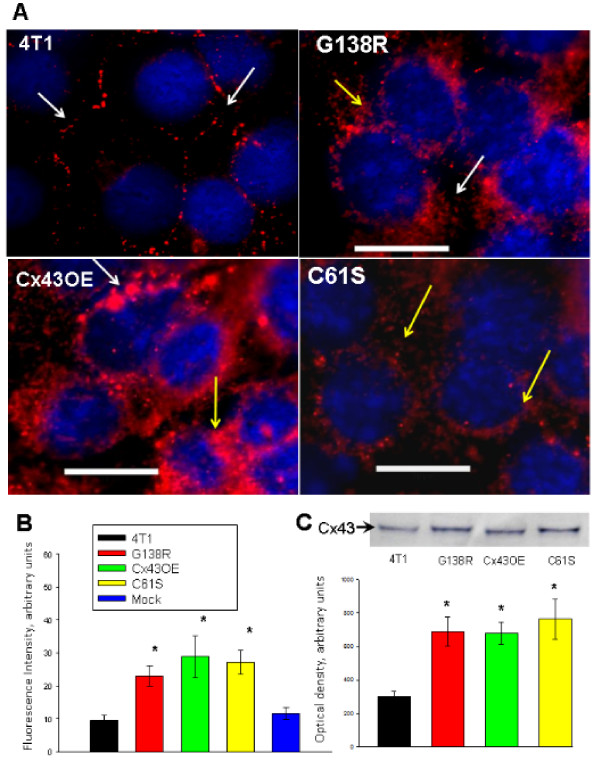
**Expression of connexin-43 by the mutant variants**. (A) Immunocytofluorescence analysis of connexin-43 (Cx43) expression in wild-type 4T1-GFP tumor cells and in 4T1-GFP cells transfected with expression vectors of the mutant G138R and C61S in addition to a vector expressing two copies of wild-type Cx43 (Cx43OE). Endogenous Cx43 in wild-type 4T1-GFP cells is present mostly at the plasma membrane between the cells (3A, 4T1, white arrows). The G138R dominant-negative mutant was expressed both at the plasma membrane (white arrows) and in the cytoplasm (yellow arrow). Wild-type overexpression led to upregulation of Cx43 in both the plasma membrane (white arrows) and in the cytoplasm (yellow arrows, 3A, Cx43OE). Cx43 with the C61S mutation was mostly in the cytoplasm (yellow arrows, 3A, C61S). (B) Quantification of the immunocytofluorescence signal of Cx43 in 4T1-GFP cells expressing mutant and wild-type Cx43. All of the transfectants showed at least a factor or two more Cx43 expression compared with endogenous expression (control) or with mock transfection. (C) Western blot analysis showing increased expression of Cx43 forms in transfected cells when compared with non-transfected 4T1-GFP cells. The quantification of bands from three blots illustrates that the transfected cells show an at least two-fold increase in Cx43 expression compared with wild-type 4T1-GFP cells. *P < 0.05.

The presence of endogenous Cx43 was demonstrated in wild-type 4T1 cells where Cx43 is localized mostly at the plasma membrane between the cells (Figure 6A, 4T1, white arrows). The G138R dominant-negative mutant as well as the Cx43 wild-type Cx43OE cell line expressed Cx43 both at the plasma membrane (white arrows) and in the cytoplasm (yellow arrows). The mutant C61S expressed a non-targetable form of Cx43 mostly in the cytoplasm (Figure 6A, yellow arrows). Quantification of the immunocytofluorescence signal of Cx43 in 4T1-GFP cells expressing mutant and wild-type Cx43 demonstrated an at least two-fold increase in Cx43 expression compared with endogenous expression (control) or with mock transfection (Figure 6B). Western blot analysis indicated increased expression of Cx43 forms in transfected cells compared with non-transfected 4T1-GFP cells (Figure 6C). The quantification of bands from three blots illustrates that the transfected cells show an at least a two-fold increase in Cx43 expression (*P *< 0.05) compared with wild-type 4T1-GFP cells (Figure 6C, bar graph).

**Figure 7:**
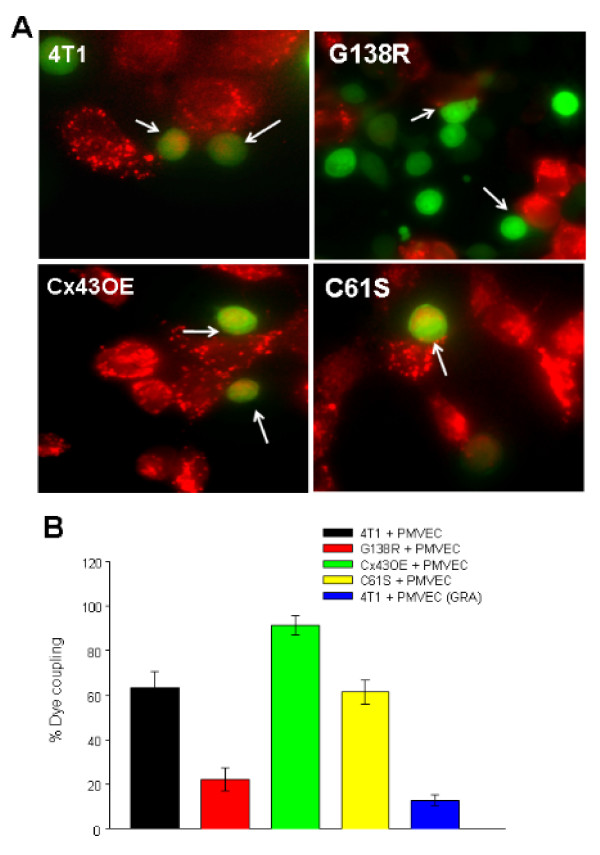
**Dye transfer between 4T1-GFP and pulmonary microvascular endothelial cells**. (A) Subconfluent monolayers of pulmonary microvascular endothelial cells in coverglass-bottomed dishes was labeled with 5 μM Calcein Red AM and then thoroughly washed for removal of extracellular dye. 4T1-GFP cells with or without connexin-43 (Cx43) mutation variants were deposited into the dish and placed in a cell culture incubator. After 1 hour, the cells were imaged in the red (for Calcein Red) and green fluorescent protein (GFP) channels and the pictures were overlaid. In tumor cells (green) that were in close contact with endothelial cells, transfer of Calcein Red can be observed (red color inside green cells). (B) Quantification of 4T1-GFP cells exhibiting dye transfer from endothelial cells. Dye transfer was significantly reduced in 4T1-GFP tumor cells expressing the G138R Cx43 mutant (red bar) and in the presence of gap junctional inhibitor 18β-glycyrrhetinic acid (30 μM) (blue bar). On the other hand overexpressing wild-type Cx43 enhanced dye transfer (green bar). The expression of C61S mutant did not seem to affect dye transfer (yellow bar).

When 4T1-GFP cells were placed on top of Calcein Red Orange labeled subconfluent monolayer of endothelial cells, the 4T1-GFP cells established functional gap junctions with the endothelial cells. In tumor cells (green of GFP) that were in close contact with endothelial cells, transfer of Calcein Orange Red from endothelial cells can be observed (red color inside green cells; Figure 7A, 4T1, and Figure 4B, black bar). Dye transfer was significantly reduced in tumor cells expressing the G138R mutant (Figure 7A, G138R, and Figure 7B, red bar) and in the presence of gap junctional inhibitor 18β-glycyrrhetinic acid (30 μM; Figure 7B, blue bar). On the other hand, overexpressing wild-type Cx43 enhanced dye transfer (Figure 7A, Cx43OE, and Figure 7B, green bar). The expression of C61S mutant did not affect dye transfer via endogenous Cx43 (Figure 7A, C61S, and Figure 7B, yellow bar).

Immunostaining revealed the presence of Cx43 at the contact area of some tumor cells that were attached to the apical surface of underlying endothelial cells (Figure 10A).

### Role of Cx43 in the metastatic efficiency

#### Cx43 facilitates tumor cell adhesion to lung endothelium

**Figure 8:**
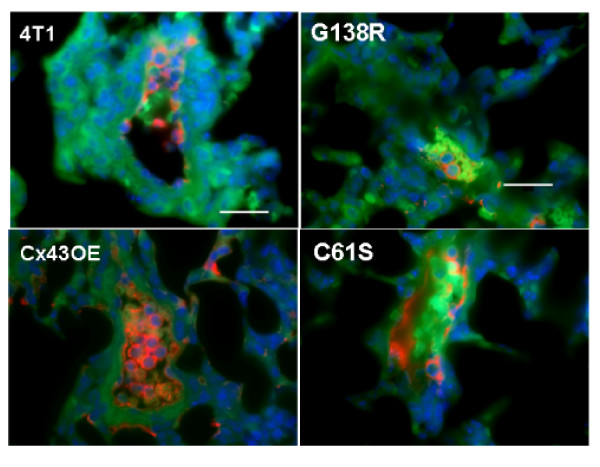
**Role of connexin-43 in tumor cell attachment to lung endothelium**. Immunofluorescence for connexin-43 (Cx43) of mouse lung sections 12 hours after tail vein injection with 4T1-GFP control cells or cells expressing Cx43 variants C61S, G138R, or Cx43OE. Autofluorescence of lung tissue and red blood cells in the green fluorescent protein (GFP) channel (green), Cx43 signal in the Cy5 channel (red), and the nuclear DAPI signal (blue) were overlaid. Panel '4T1' (control 4T1-GFP): Cx43 (red) was upregulated in the tumor cells and in the endothelium of the vessels with tumor cells. Panel 'C61S': tumor cells with C61S mutation, showing similar level of Cx43 expression as for control cells. Panel 'G138R': cells with dominant negative G138R mutation, showing Cx43 (red) around a few tumor cells within the vessels with a limited number of tumor cells in the vessels. In contrast lung segments containing 4T1 tumor cells overexpressing Cx43 show intense Cx43 staining and the vessels contained large number of tumor cells (see panel 'Cx43OE'). The scale bar is 25 μm for all panels.

**Figure 9:**
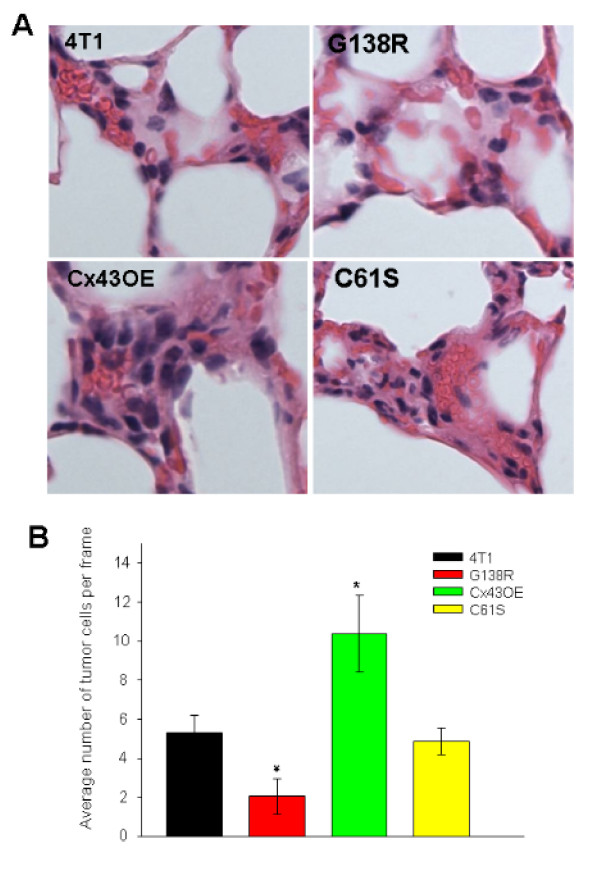
**Connexin 43 facilitates tumor cell attachment to lung endothelium**. (A) Hematoxylin-eosin staining of mouse lung sections 12 hours after tail vein injection with 4T1-GFP control cells or cells expressing connexin-43 (Cx43) variants C61S, G138R, or Cx43OE. Panel '4T1': a vessel containing red blood cells and dark nuclei of adherent 4T1-GFP control cells within it. Panel 'C61S': C61S expressing tumor cells show a comparable density of tumor cell nuclei in blood vessels. Panel 'G138R': dominant-negative G138R expressing tumor cells show a decreased number of tumor cells inside vessels. In contrast, tumor cells overexpressing Cx43 appear to populate the vessels in much larger number (see panel 'Cx43OE'). (B) Quantification of the tumor cell numbers adhering to the lung endothelium showing that dominant-negative expression Cx43 reduced the number of the tumor cells within the vessels in the lung (red bar), while overexpressing wild-type Cx43 resulted in more tumor cells within the lung vasculature (black bar). *P < 0.05 versus 4T1. The results represent three lungs for each condition, four slides per lung, and 20 contiguous picture frames per slide.

A role for Cx43 in tumor cell attachment to the pulmonary endothelium *in vivo *is demonstrated by the following observations. Cx43 expression, detected by immunofluorescence (red pseudocolor), was upregulated in the tumor cells and in the endothelium of the vessels with control tumor cells (Figure 8, 4T1). Tumor cells with C61S mutation showed similar levels of Cx43 expression in the endothelium as for control cells (Figure 8). In both cases, the number of arrested cells within the vasculature was similar. Fewer cells with the G138R mutation were found arrested (Cx43 signal in red; Figure 8). In contrast, lung vasculature containing 4T1-Cx43OE tumor cells showed intense Cx43 staining of the endothelium and the tumor cells (Figure 8). There were also significantly more tumor cells in the blood vessels of these lungs. These data indicate a cell adhesion role for functional Cx43 in breast cancer metastasis to the lung in this syngeneic model. Similar results were obtained with immunohistochemical analysis of lung tissue sections containing metastatic tumors of the variant Cx43 4T1-GFP cell lines. Figure 9A, 4T1, depicts a vessel containing red blood cells and dark nuclei of adherent control 4T1-GFP cells within it. The number of vessel-adherent tumor cells expressing the C61S mutation was comparable to that for control cells (Figure 9A). On the other hand, the number of adherent tumor cells with the G138R mutated Cx43 was significantly lower (Figure 9A, G138R). Tumor cells overexpressing Cx43 appear to populate the vessels in much greater numbers (Figure 9A, Cx43OE). Quantification of the tumor cell numbers adhering to the lung endothelium shows that dominant negative G138R expression reduced the number of arrested tumor cells (Figure 9B, red bar), while overexpressing wild-type Cx43 increased the number of arrested tumor cells within the lung vasculature (Figure 9B, green bar).

## Discussion

Although a variety of connexins are involved in GJIC in different cell types, the expression of Cx43 in vascular endothelial cells and in many tumor cell lines indicates a primary role for this protein in the heterologous gap junctional communication during tumor cell-endothelial cell interaction [23-28]. Functional GJIC between tumor cells and vascular endothelial cells mediated via Cx43 has been demonstrated [9,23,25]. Consequently, it was also shown that quantitative and qualitative changes in connexin expression are associated with tumor progression, proliferation, invasion, and metastasis [9,25,29]. Cx43 enhanced the adhesiveness and mediated the invasion in malignant gliomas [20]. In addition, the increased expression of specific connexins correlated with increased invasiveness of lung squamous cell carcinoma or NIH3T3-Ras cells [29,30] and Cx43 expression was upregulated in urethane-induced mouse lung adenomas [31]. Increased levels of Cx43 mRNA was found in hepatocellular carcinoma tissues [32] and in the stroma of multiple intestinal neoplasia-mice adenomas [23]. Expression of Cx43 in mammary carcinoma cell lines lacking endogenous Cx43 enabled the formation of heterocellular GJIC with microvascular endothelial cells and increased their diapedesis [12]. Increased cytosolic and plasma membrane expression of Cx43 in lymph node metastases of breast cancer was demonstrated [11]. Cx43 was detectable in normal lung, smaller size tumor, and larger size mouse lung tumors [24]. Our study shows a role for Cx43 in breast cancer metastasis to the lung in a syngeneic, murine experimental metastasis model.

Although connexins are classically considered as gap-junctional proteins, non-junctional roles for connexins in hemichannels with ion-channel-like functions or possessing channel-independent, signaling-type functions have received considerable attention recently [33,34]. Adhesion-mediated establishment of functional GJIC between lung-metastatic B16F10 melanoma cells and endothelium has been shown to be dependent on the expression of Cx43 in both cell partners [35].

**Figure 10:**
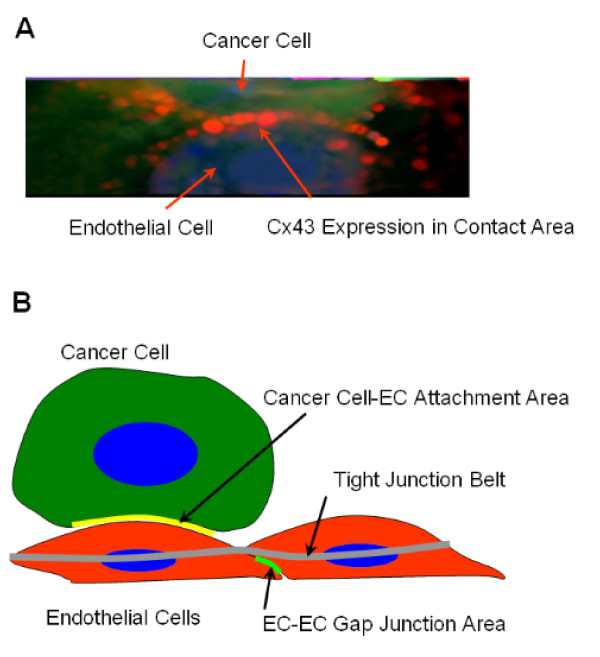
**Apical endothelial cell connexin 43 forms heterologous gap junctions**. (A) Connexin-43 (Cx43) localization at the interface between the endothelial apical surface and the 4T1-GFP cell coupling surface. The picture shows a side view of a 4T1-GFP cell sitting on top of an endothelial cell in culture. The side view was generated from a three-dimensional reconstructed image from a stack of 20 *z*-axis optical sections of the cells. (B) Model of cancer cell attachment to endothelial cells. The gap junctional coupling mediated via Cx43 would require its localization on the surfaces in contact: the apical surface of the endothelial cells and the coupling surface of the cancer cell. Our results suggest that the initial attachment mediated via the classical cell adhesion molecules (such as α_3_β_1 _integrin and vascular laminin-5 [42]) may not be sufficient for retention of the cells and the creation of metastatic foci. This requires establishment of functional gap junctions between the apposing surfaces brought together by cell adhesion molecules. In our model, the required molecule playing an important role in the adhesion efficiency is Cx43, as demonstrated by the loss of adhesion with gap junctional intercellular communication incompetent G138R mutant variant in the cancer cell alone. How the communication helps the establishment of the metastatic foci is unknown at present, but we speculate that the coordination between the disparate cell types helps them to recognize each other as partners and makes them grow together to become a tumor mass with blood supply.

The major conclusion of this study is that gap junctional communication via Cx43 facilitates metastatic homing by increasing the arrest of cancer cells in the lung vasculature. The reduced attachment of cancer cells with the dominant-negative G138R that allows formation of gap-junction plaques but not of functional junctions suggests a positive correlation between the loss of intercellular communication and the number of adherent tumor cells in the lungs. The idea that the formation of functional gap junctions between a cancer cell and the endothelial cell is critical for tumor cell adhesion to the pulmonary endothelium is novel. Normal gap junction plaques are found in the lateral membranes of cells, below the level of the tight junction belt. In the case of heterologous gap junction formation with adherent cells, the apical surface of the endothelial cells comes into contact with the surface of cancer cells (Figure 10). Therefore, a role for connexins in heterologous adhesion or in establishment of functional GJIC would require the presence of connexins at the apical surface of endothelial cells, as demonstrated for Cx43 in Figure 10A. Very high concentration of Cx43 has been demonstrated at the apical surface of epithelial cells [36]. The residual coupling between Cx43G138R expressing tumor cells and endothelial cells expressing wild-type Cx43 in comparison to 18β-glycyrrhretinic acid treated cells (Fig 7B red and blue bars) is mediated through heterotypic Cx43G138R-Cx43WT channels. These channels were recently described as strongly inhibited but not completely abolished in their function [37]. The decreased attachment efficiency with the G138R mutation might also be the result of alteration in conformation of the Cx43 protein. The C61S mutant does not express the mutated Cx43 in the membrane; therefore, it did not alter the cell adhesion mediated by endogenous Cx43. The increased adhesion of cancer cells with overexpression of wild-type Cx43 further confirms the role for Cx43 in tumor cell attachment efficiency as a result of enhanced GJIC.

The marked upregulation of Cx43 in tumor cell-endothelial cell contact areas, whether in preexisting 'homing' vessels or in newly formed tumor vessels, indicates that Cx43 can serve as a marker of micrometastases and tumor vasculogenesis. This raises the possibility of a role for Cx43 in the early incorporation of endothelial cells into pre-hypoxia size tumors as seeds for vasculogenesis.

Cx43 has been shown to enhance angiogenesis *in vitro *[38]. Tube formation by human umbilical vein endothelial cells cocultured with Cx43-transfected malignant glioma cells or with naturally Cx43-expressing malignant glioma cells was significantly increased compared with tube formation by endothelial cells alone [5]. In addition, upregulation of Cx43 in the hypertrophic myocardium in mice suggested its role in cardiac angiogenesis [38]. These reports are consistent with our finding of upregulation of Cx43 in cancer cell-endothelial cell contact areas. This selective upregulation of Cx43 can be exploited as a marker for sites of intravascular metastases as well as for vasculogenic loci within metastatic tumors. Previously, it had been shown that Cx43 mRNA in normal tissue surrounding lung tumor may act as a molecular marker of nodal micrometastasis in non-small cell lung cancer [39].

The association between cancer and the lack of communication between cancer cells set the stage for a role of dysfunctional gap junctions in carcinogenesis nearly 40 years ago [40]. Since then, both increased and decreased levels of various connexins shown in different models of primary and metastatic tumors had generated multiple interpretations [41]. The difficulty in developing a unified hypothesis regarding the role of connexins in primary and secondary carcinogenesis may be associated with a multiplicity of contradictory functions that the metastatic cell has to perform during intravasation and distant localization. A recent review predicted that "a gain of function (of gap junctions) may characterize the metastatic stage" [41]. Our results confirm this prediction and we hope that they will set the stage for a renewed assessment of the role of connexins in metastatic tumorigenesis and tumor vasculogenesis.

## Conclusion

Cx43 facilitates metastatic 'homing' by increasing adhesion of cancer cells to the lung endothelial cells. The marked upregulation of Cx43 in tumor cell-endothelial cell contact areas, whether in preexisting 'homing' vessels or in newly formed tumor vessels, suggest that Cx43 can serve as a potential marker of micrometastases and tumor vasculature and that it may play a role in the early incorporation of endothelial cells into small tumors as seeds for vasculogenesis.

## Competing interests

The authors declare that they have no competing interests.

## Authors' contributions

MKE carried out the fluorescence imaging, immunofluorescence, experimental metastatic tumor development in the nude mice, organized the data, and drafted the manuscript. AH generated experimental metastatic tumors in the nude mice. KW generated the C61S and Cx43OE constructs. RD generated the G138R construct and revised the manuscript. MNG participated in the design of the study and revised the manuscript. ABA conceived of the study, participated in its design and coordination, drafted and revised the manuscript. All authors have read and approved the manuscript.

## Pre-publication history

The pre-publication history for this paper can be accessed here:


